# The graded effect of propofol in electrophysiology-guided navigation during deep brain stimulation surgery

**DOI:** 10.1038/s41531-025-01243-1

**Published:** 2026-01-29

**Authors:** G. Issabekov, B. Al-Fatly, M. Mousavi, J. Roediger, R. Köhler, J. Habets, A. L. de Almeida Marcelino, M. S. Tuncer, P. Krause, M. Astalosch, D. Kübler-Weller, C. Spies, P. Spindler, K. Faust, P. Truckenmueller, G. H. Schneider, A. A. Kühn, L. A. Steiner

**Affiliations:** 1 Movement Disorder and Neuromodulation Unit, Department of Neurology and Experimental Neurology, Charité - Universitätsmedizin, Berlin, Germany; 2 Technische Universität, Berlin, Germany; 3 Berlin Institute of Health (BIH), Berlin, Germany; 4 Department of Anesthesiology and Intensive Care Medicine, Campus Charité Mitte and Campus Virchow-Klinikum, Charité - Universitätsmedizin, Berlin, Germany; 5 Department of Neurosurgery, Charité - Universitätsmedizin, Berlin, Germany; 6 Neurosurgical Clinic, Heinrich-Heine-University, Düsseldorf, Germany

**Keywords:** Medical research, Neurology, Neuroscience

## Abstract

Propofol is widely used for general anesthesia (GA) during deep brain stimulation (DBS) surgery targeting the subthalamic nucleus (STN) in Parkinson’s disease (PD), yet its effects on intra-operative spatial navigation, critical for electrode placement, remain contentious. We performed multimodal analysis on 583 microelectrode recordings (MER) from PD patients undergoing DBS surgery under local anesthesia (LA) and GA. Deep sedation interfered with the identification of the dorsal STN border, and propofol dosages >4 mg/kg/h resulted in deeper final electrodes. While firing rate (FR) and burst index (BI) differed between LA and GA, only BI distinguished imaging-defined STN and correlated negatively with the proximity to the DBS sweetspot across conditions. Thus, propofol-based GA complicates navigation in DBS surgery, but MER remain informative if propofol levels are carefully controlled. BI emerges as a potential biomarker when MER are “polluted” by high levels of propofol, offering critical feedback during DBS surgery under GA.

## Introduction

Deep brain stimulation (DBS) surgery targeting the subthalamic nucleus (STN) is an established stereotactic neurosurgical intervention for the treatment of motor symptoms in Parkinson’s disease (PD). Surgical precision is a critical determinant of achieving optimal DBS-mediated improvements in motor performance^1^. Microelectrode recordings (MER) are performed along the anatomically planned trajectory to confirm target location, provide spatial orientation by identifying patient-specific borders of target structures, and allow for clinical assessment of the therapeutic windows at specific depths along the trajectory^2^. The information obtained during MER critically informs the neurosurgeon’s decision regarding final placement of the permanent electrode.

In recent years, DBS surgery has shifted from being performed on fully awake patients with local anesthesia (LA) to patients being operated on under general anesthesia (GA) in many centers^3,4^. This transition has been driven by advances in preoperative imaging, reductions in surgery time and costs, patient preferences and efforts to address the intra-operative challenges of awake surgery, particularly those concerning patient management^5^. However, sedative agents significantly alter electrophysiological fingerprints at both neuronal and oscillatory level, potentially compromising the identification of the dorsal STN border and differentiation of the STN from the Subtantia nigra pars reticulata (SNr). These concerns have sparked an ongoing debate regarding optimal anesthesia protocols for DBS surgery^6^.

Propofol, a gamma-Aminobutyric acid (GABA)-A agonist, is the most frequently used sedative agent due to its rapid metabolism, safety, and ease of titration^7^. Several studies have demonstrated that STN identification remain feasible under propofol-based GA^8–12^. Nevertheless, neuronal firing has been reported to be altered during propofol administration in a dose- and time-dependent manner^13^. Specifically, STN neurons exhibit reduced firing rates and excessive burst discharge characteristics under propofol sedation^14^.

Despite the aforementioned insights into the effects of propofol on neuronal firing patterns, its influence on MER in the spatial domain remains unclear. The decision regarding final electrode placement is made within patient-specific anatomical space, best characterized by integrating preoperative imaging and high-resolution intra-operative electrophysiology. Thus, understanding how propofol alters neuronal feature topologies may have direct relevance for clinical decision-making.

In this single-center, retrospective observational study, we analyzed 702 neuronal recordings from 25 patients who underwent STN-DBS implantation and localized MER into a common anatomical space based on postoperative electrode reconstructions. We related neuronal features including firing rate (FR), burst index (BI), and coefficient of variation (CV) to intra-operative electroencephalography (EEG), enabling an objective assessment of sedation depth independent of patient-specific drug metabolism. Furthermore, we leveraged the variability in intra-operative propofol levels to stratify the role of electrophysiology in DBS implantation surgeries performed under GA. Building on our spatial analysis, we examined how neuronal features such as the BI were distributed across patients within a common space and performed correlative analyses to assess the relationship between these neuronal features and pre-defined DBS sweetspot under both LA and GA. Finally, we compared final electrode placements in DBS surgeries performed under LA versus GA to evaluate the clinical implications of the anesthetic regime applied.

Ultimately, this study demonstrates that despite GA-induced alterations in single-unit activity topologies, these neuronal features can still provide informative guidance during DBS surgery.

## Results

**Fig. 1:**
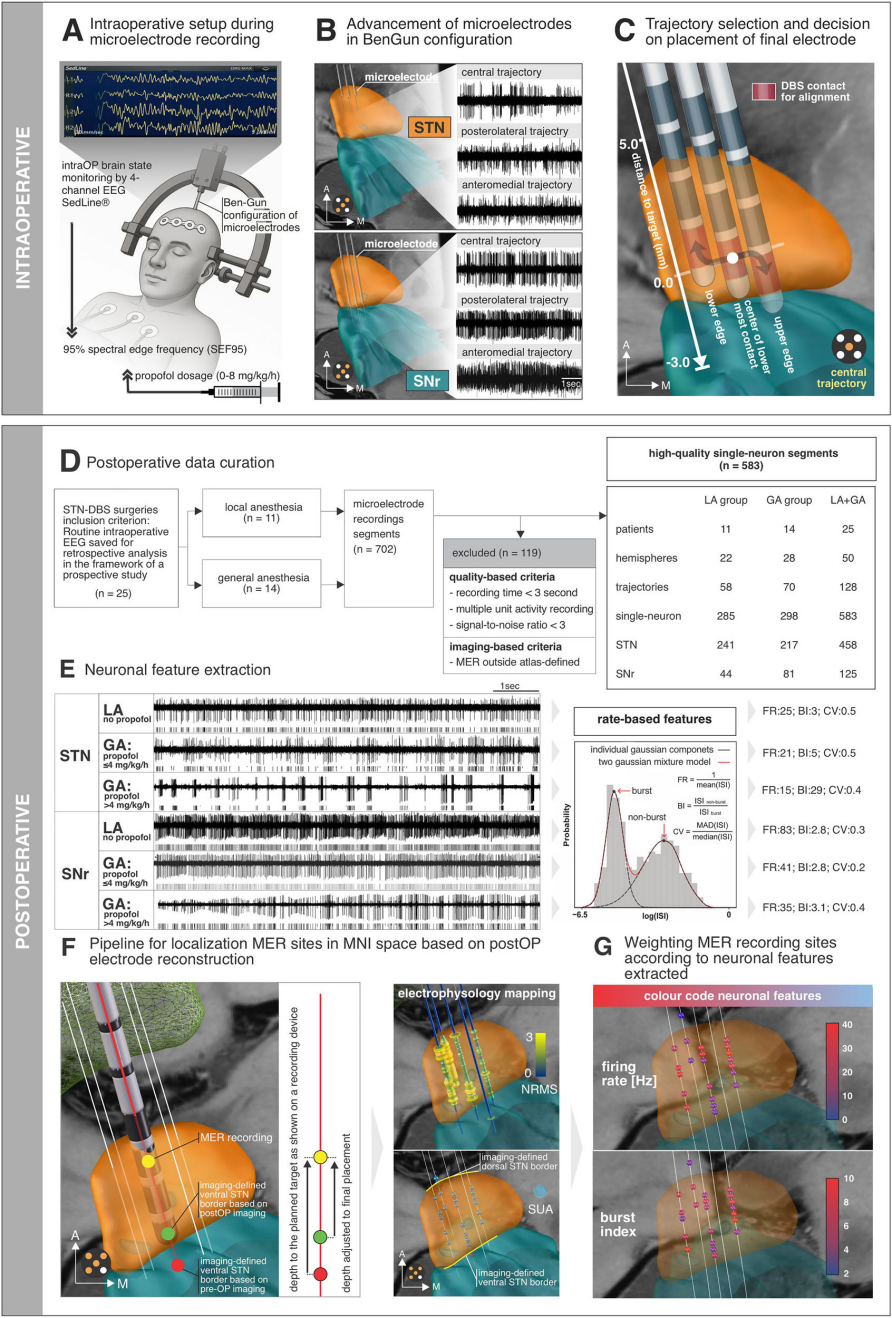
Overview of intra-operative and postoperative pipeline for mapping neuronal activity acquired during DBS lead implantation surgery. **A** Schematic of intra-operative setup during DBS surgery, including Ben Gun array to hold microelectrodes, frontal EEG monitoring (4-channel SedLine® system), and sedation titration with propofol. Spectral edge frequency at 95% (SEF95) was extracted from intra-operative EEG to quantify depth of sedation. **B** Microelectrodes were advanced along multiple trajectories in a Ben-Gun configuration (central, posterolateral, anteromedial), traversing the STN and SNr. Corresponding electrophysiological activity is shown for each trajectory. **C** Final electrode placement was guided by considering anatomical planning, intra-operative electrophysiology and testing for side effects as elicited by test stimulation. **D** Flowchart outlining data curation from 25 STN-DBS surgeries. A total of 702 neuronal segments were recorded, of which 583 high-quality single-unit activity recordings were included after quality- and anatomy-based filtering. **E** Examples of neuronal recordings from STN and SNr under local anesthesia (LA) and general anesthesia (GA), including high-dose propofol (>4 mg/kg/h) and low-dose propofol (≤4 mg/kg/h) conditions, illustrating differences in spiking activity. Right: method for extracting firing rate (FR), burst index (BI), and coefficient of variation (CV) using a two-component Gaussian mixture model of log (ISI) distribution. **F** Pipeline for localizing MER sites in standardized MNI space using postoperative electrode reconstructions. Left: adjusting depth of MER site along the trajectory of final electrode based on intra-operative electrode positioning. Right: mapping of neuronal features along reconstructed trajectories. For spatial visualization, normalized root mean square (NRMS) was depicted using a tubular structure, with the radius reflecting NRMS magnitude, while SUA was represented as spheres. **G** Spatial visualization of MER sites. Top: FR; bottom: BI. Color coding reflects relative feature magnitude at each recording site as outlined by color bar.

This retrospective study included 25 patients (11 female, 60 ± 6 years (mean ± std) at surgery, demographics available in Supplementary Table 1), with 11 in the local anesthesia (LA) group and 14 in the general anesthesia (GA) group (Fig. 1D). A total of 128 microelectrode trajectories were analyzed (58 in LA and 70 in GA), yielding 583 high-quality single units (285 in LA and 298 in GA) within either the STN or the SNr.

### High propofol levels risk missing STN entry

**Fig. 2:**
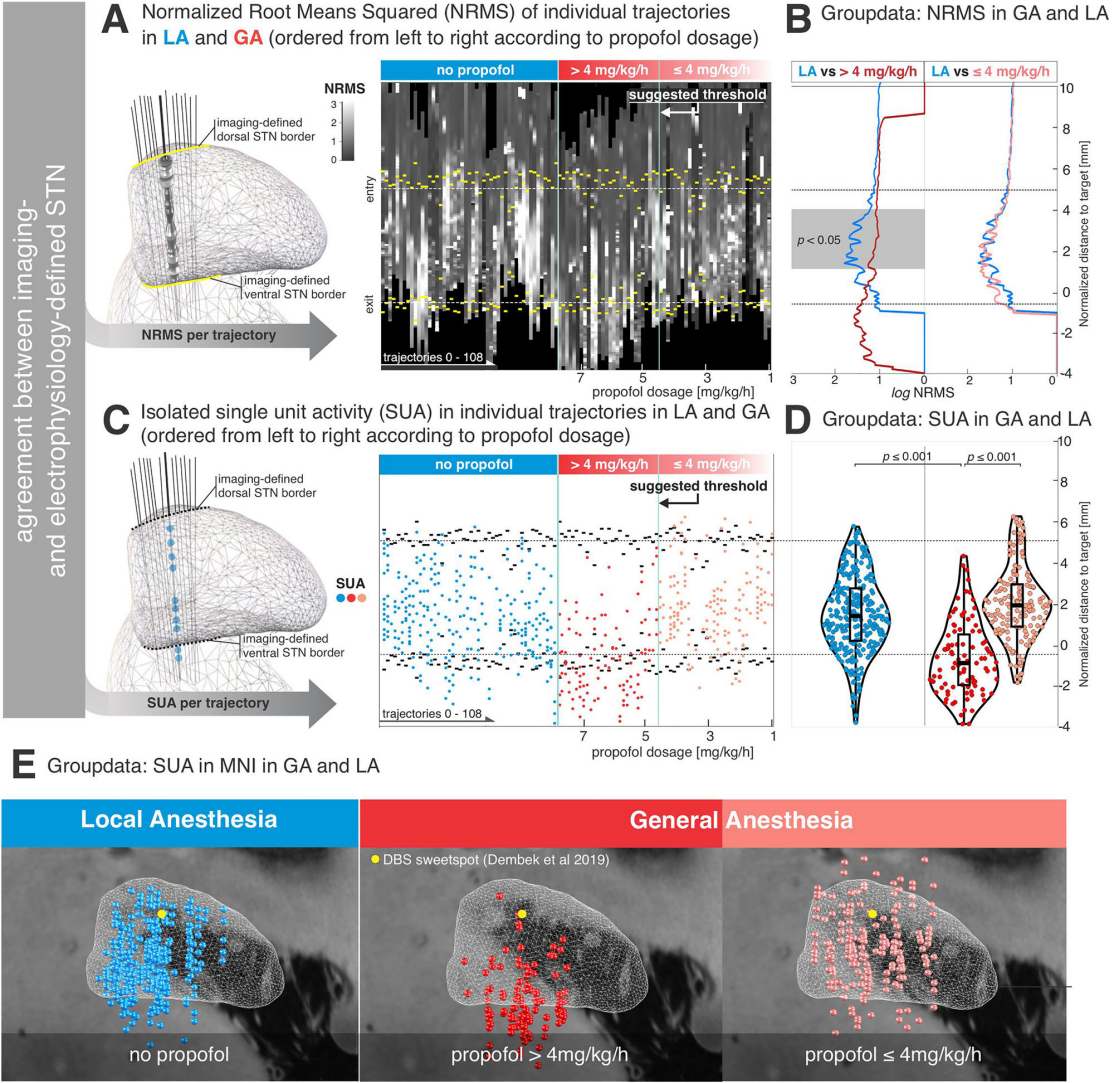
Effects of propofol sedation on background unit activity and single-unit spiking along the dorsoventral STN axis. **A** NRMS activity per trajectory under local anesthesia (LA) and general anesthesia (GA). GA trajectories are sorted by propofol dosage and stratified into high (>4 mg/kg/h) and low (≤4 mg/kg/h) subgroups, aligned to the imaging-defined STN midpoint. **B** Group-level comparison of NRMS across conditions. A cluster-based permutation test showed significantly lower NRMS in high-dose GA (> 4 mg/kg/h) compared with LA (*p* < 0.05, FDR-corrected), within the STN (gray). No significant difference appeared between LA and low-dose GA (≤ 4 mg/kg/h). Group sizes: LA, *n* = 49; GA > 4 mg/kg/h, *n* = 27; GA ≤ 4 mg/kg/h, *n* = 30. Lines indicate group medians. **C** Depths of isolated SUA under LA and GA, sorted by propofol dose and aligned to the imaging-defined STN midpoint. **D** Violin plots of global SUA depths. High-dose propofol caused a dorsal shift in SUA occurrence, suggesting impaired detection of the dorsal STN border. **E** Spatial distribution of SUA in MNI space. Statistical analysis of MNI x, y, z coordinates showed significant group differences (*p* < 0.001), most prominently along the z-axis across LA, GA > 4 mg/kg/h, and GA ≤ 4 mg/kg/h (Supplementary Fig. 1). Clinical DBS sweetspot according to Dembek et al., 2019.

To visualize the occurrence of neuronal activity, trajectories recorded under GA were sorted by propofol infusion rate applied during MER (range: 1.0–7.5 mg/kg/h; mean ± std: 3.9 ± 1.8 mg/kg/h). This revealed a threshold-like relationship between the propofol level and suppression of neuronal activity, with a notable reduction emerging around ~4 mg/kg/h (Fig. 2). A cluster-based permutation test revealed a significant difference in normalized root mean square (NRMS) values between conditions over a specific range of distances. Notably, the group-level average aligned distance to target—STN entry point was 5.0536 mm above target, while the average exit point was 0.5591 mm below target. A significant cluster was identified for LA vs. GA (propofol >4 mg/kg/h) between 3.6 mm and 1.2 mm above target (*p* < 0.05, corrected), characterized by higher NRMS values in the LA group (Fig. 2A, B). In contrast, no significant cluster was found for LA vs. GA (propofol ≤4 mg/kg/h) groups within the STN (Supplementary Fig. 3). Next, we compared the depths of isolated SUA along the included trajectories across three conditions - LA, GA (propofol >4 mg/kg/h) and GA (propofol ≤4 mg/kg/h) (Fig. 2C, D). ANOVA revealed a significant main effect for condition F_2,527_ = 68.85, *p* ≤ 0.001, d = 1.02, posthoc power estimate = 0.9998. SUA in the GA group receiving high dosage of propofol >4 mg/kg/h (– 0.07 ± 2.38, mean ± std; *n* = 96) occurred deeper along the trajectory compared to both the LA group (2.32 ± 3.01, mean ± std, *n* = 285; *p* < 0.001) and the GA group receiving propofol ≤4 mg/kg/h (2.39 ± 2.94, *n* = 148; *p* < 0.001). No significant difference was found between the LA group and the GA ≤ 4 mg/kg group (*p* > 0.99). We quantified spatial separation between groups (Fig. 2E) using Euclidean distances in MNI space across the x, y, and z coordinates and evaluated these differences using MANOVA (Supplementary Fig. 1). The multivariate test revealed significant inter-group differences (Wilks’ Λ = 0.71, F_6,569_ ≈ 17.57, *p* < 0.001, d = 0.86, posthoc power estimate >0.99). Post hoc analyses revealed significant pairwise differences (*p* < 0.001), most prominently along the z-axis across three conditions.

### Burst index, but not firing rate or coefficient of variance, discriminates STN from SNr in LA and GA

**Fig. 3:**
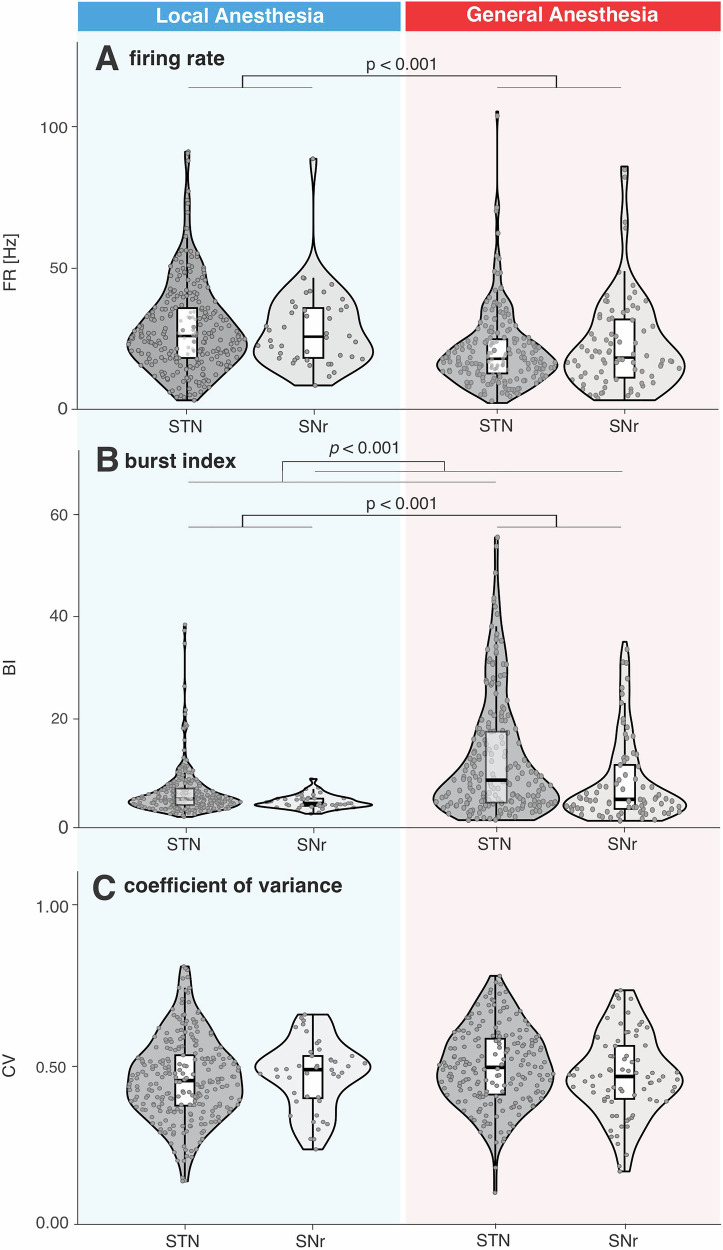
Neuronal features in imaging-defined STN and SNr under local and general anesthesia. Violin plots comparing neuronal features between imaging defined STN and SNr under local anesthesia (LA) and general anesthesia (GA). A total of 301 neurons (257 STN, 44 SNr) under LA and 282 neurons (201 STN, 81 SNr) under GA were analyzed. Box plots indicate the median and interquartile range. See results for detailed report of statistics. FR Firing rate, BI Burst index, CV Coefficient of variance.

To examine the impact of different anesthesia conditions on rate-based neuronal features (FR, BI, CV), comparisons were performed between the STN and SNr under LA and GA. Figure 3 presents the analysis of these features within the imaging-defined STN and SNr. To avoid circularity, we considered the imaging-defined STN and SNr for statistical analysis. Comparison of neuronal features of STN and SNr as defined by the intra-operative electrophysiologist are shown in the Supplementary Fig. 5. Analysis of variance (ANOVA) of firing rate for imaging defined STN and SNr revealed a significant main effect for anesthesia (F_1,579_ = 21.27, *p* < 0.001, d = 0.384; posthoc power estimate = 0.996), but no main effect of structure (F_1,579_ = 0.34, *p* = 0.559, d = 0.05, posthoc power estimate = 0.09) or structure*anesthesia interaction (F_1,579_ = 0.63, *p* = 0.427, d = 0.07, posthoc power estimate = 0.13). The main effect for anesthesia was due to higher firing rates in LA (28.47 ± 14.00 Hz, mean ± std; *n* = 301) compared to GA (21.07 ± 14.37 Hz, mean ± std; *n* = 282). Analysis of variance for burst index for imaging defined STN and SNr revealed a significant main effect for anesthesia (F_1,580_ = 54.95, *p* < 0.001, d = 0.62, posthoc power estimate = 0.9999), a significant main effect of structure (F_1,579_ = 10.77, *p* < 0.001, d = 0.27, posthoc power estimate = 0.91) but no main effect of structure*anesthesia interaction (F_1,579_ = 1.39, *p* = 0.239, d = 0.10, posthoc power estimate = 0.22). The main effect for anesthesia was due to higher BI values in the GA group (11.98 ± 9.53, mean ± std; *n* = 282) compared to the LA group (5.59 ± 4.58; *n* = 301). The main effect for structure was due to higher BI values in STN (9.27 ± 8.80; *n* = 458) compared to SNr (6.58 ± 5.80; *n* = 125).

Analysis of variance for the coefficient of variation (CV) revealed no significant main effects of structure (F_1,579_ = 1.51, *p* = 0.312, d = 0.10, posthoc power estimate = 0.23), condition (F_1,579_ = 0.21, *p* = 0.521, d = 0.04, posthoc power estimate = 0.07), or structure * condition interaction (F_1,579_ = 0.51, *p* = 0.368, d = 0.06, posthoc power estimate = 0.11).

### Objective sedation depth as indexed by intra-operative EEG correlates with accuracy of dorsal STN border differentiation and neuronal features

**Fig. 4:**
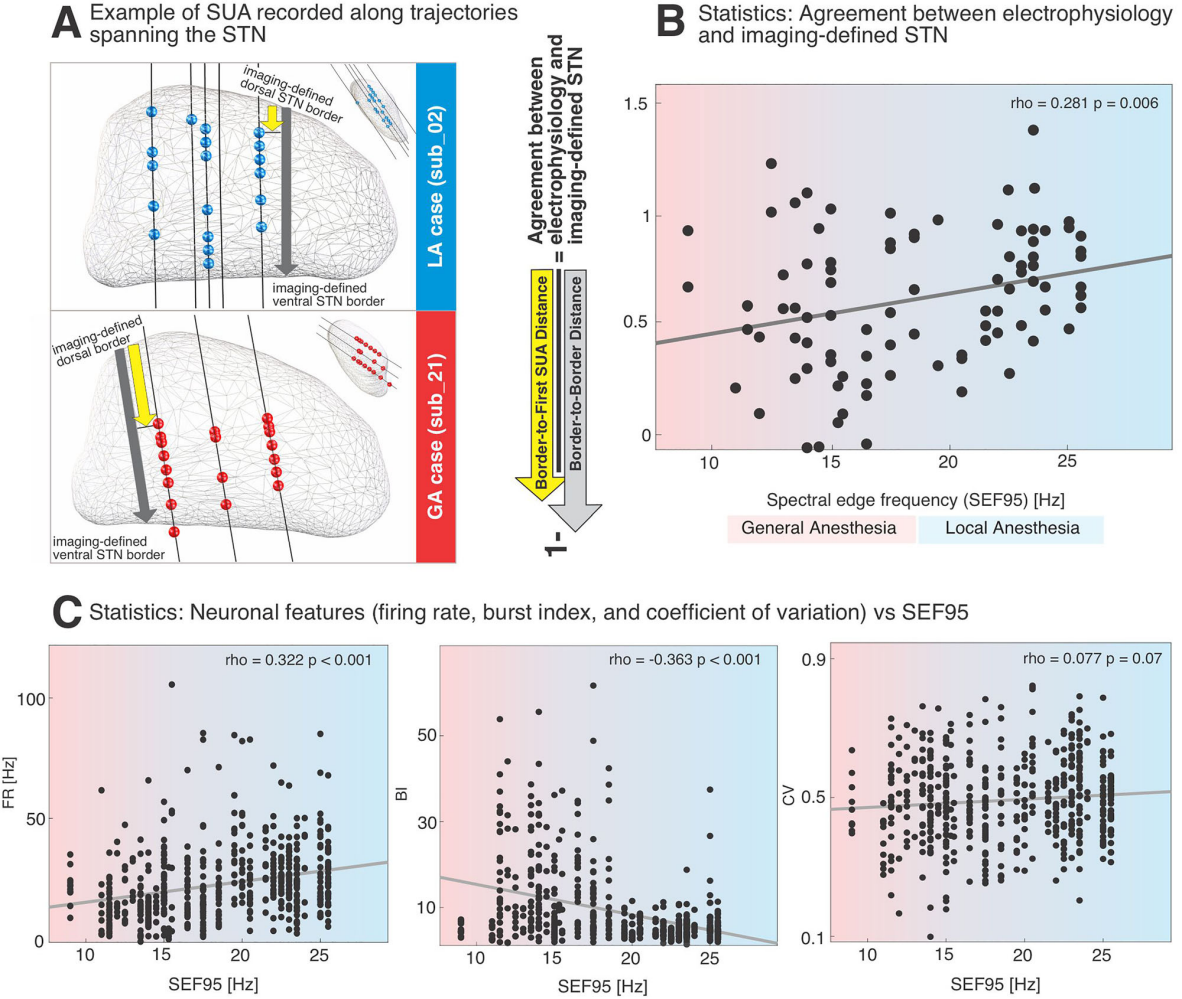
The degree of sedation, as indexed by intra-operative EEG spectral edge frequency (SEF95), correlates with the depth of the first single-unit activity relative to the imaging-defined dorsal STN border. **A** Representative trajectories from a local anesthesia (LA) case (Case 02, top) and a general anesthesia (GA) case (Case 21, bottom). In the LA case, SUA recordings span the entire STN indicating agreement between imaging- and electrophysiology-defined STN. In the GA case, three trajectories reach the STN, but SUA is absent in the dorsal region, suggesting impaired detection of the STN entry under deep sedation and insufficient agreement between imaging- and electrophysiology-defined STN. **B** Scatter plots showing the relationship between sedation depth (SEF95 from intra-operative EEG) and agreement between imaging- and electrophysiology-defined STN as derived from the relative distance from the dorsal STN border to the first isolated SUA, normalized by the length of STN traversal. Each dot represents one trajectory. A significant positive correlation was observed, indicating that lower sedation (higher SEF95) is associated with better identification of the STN entry. **C** Scatter plots showing the relationship between neuronal features (FR, BI, CV) and SEF95. Each dot represents a single neuron. Regression lines and correlation coefficients are shown for each relationship. Left: Firing Rate (FR) positively correlated with SEF95, indicating lower firing rates at deeper sedation levels. Middle: Burst Index (BI) negatively correlated with SEF95, indicating increased burstiness at deeper sedation levels. Right: Coefficient of Variation (CV) showed no significant correlation with SEF95.

There was a positive correlation between SEF95 and the normalized distance of the first SUA from the dorsal STN border (rho = 0.281, *p* = 0.006, d = 0.60, posthoc power estimate = 0.999) (Fig. 4). FR was positively correlated with SEF95 (rho = 0.322, *p* < 0.0001, d = 0.68, posthoc power estimate = 0.9999), while BI showed a negative correlation with SEF95 (rho = −0.363, *p* < 0.001, d = 0.78, posthoc power estimate = 0.9999). There was no significant correlation between SEF95 and CV (rho = 0.077, *p* = 0.07).

### Proximity to DBS sweetspot correlates with burst index in LA and GA

**Fig. 5:**
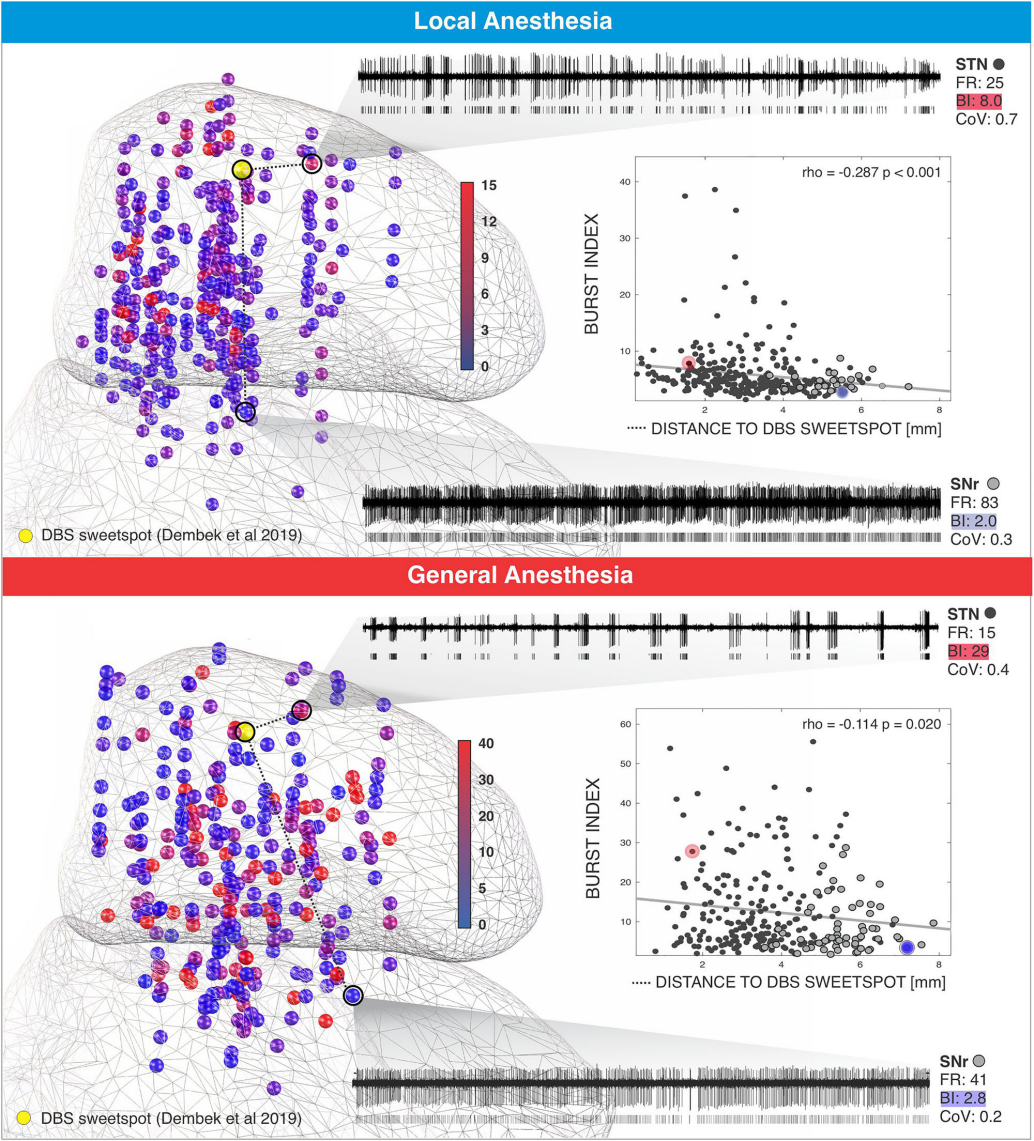
Burst index (BI) is negatively correlated with proximity to the pre-defined DBS sweetspot under both local and general anesthesia conditions. Left panels: Three-dimensional anatomical mapping of SUA recorded intra-operatively from STN and SNr across both anesthesia conditions. Each sphere represents one neuron color-coded according to its BI. A total of 301 neurons (257 STN, 44 SNr) were recorded under local anesthesia (LA), and 282 neurons (201 STN, 81 SNr) under general anesthesia (GA). Example spike trains are shown for representative STN and SNr neurons in each condition, annotated with FR, BI, and CV. Right panel: Scatter plots showing the relationship between BI and the Euclidean distance of each neuron to the DBS sweetspot. STN neurons are shown in black and SNr neurons in gray. In both LA and GA groups, BI exhibits a statistically significant negative correlation with the distance to the DBS sweetspot.

There was a negative correlation between BI and distance to the DBS sweetspot^15^ in both LA (*n* = 301, rho = −0.278, *p* < 0.001, d = 0.58, posthoc power estimate = 0.998) and GA (*n* = 282, rho = −0.114, *p* = 0.021, d = 0.23, posthoc power estimate = 0.48) groups (Fig. 5).

### High propofol doses (>4 mg/kg/h) result in deeper final electrode placement

**Fig. 6:**
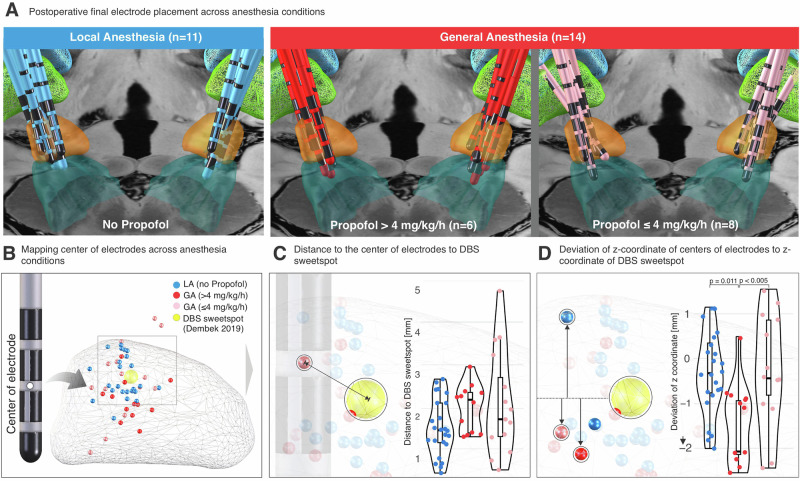
Deep sedation during MER impairs DBS lead targeting relative to the estimated clinical sweetspot. **A** Visualization of final electrode placements for patients under local anesthesia (LA, top row, blue) and general anesthesia (GA, middle and bottom rows, red and pink), stratified by intra-operative propofol dosage (>4 mg/kg/h and ≤4 mg/kg/h). Postoperative electrode reconstructions are overlaid on anatomical templates with STN (orange) and SNr (cyan) structures. **B** 3D rendering of all electrode centers mapped in MNI space relative to the clinical DBS sweet spot (yellow sphere; Dembek et al., 2019). Dots represent the center of the electrode, color-coded by condition. **C** Violin plots showing the Euclidean distance from each electrode center to the DBS sweet spot. Electrodes implanted under propofol >4 mg/kg/h show a trend toward greater displacement from the optimal target compared to LA and ≤4 mg/kg/h conditions. **D** Violin plots displaying the z-coordinate deviation between the center of the electrode and the sweet spot’s z-coordinate. High-dose propofol (>4 mg/kg/h) was associated with a significant deviation along the z-axis, suggesting compromised depth targeting under deep sedation.

ANOVA did not reveal a significant main effect when comparing the distance of final electrodes to the DBS sweetspot^15^ (F_2,47_ = 2.16, *p* = 0.127, d = 0.61, posthoc power estimate = 0.38). Separate ANOVA showed a significant difference when comparing the deviation of z-coordinates of the centers of final electrodes from the z-coordinate of the DBS sweetspot^15^ (F_2, 47_ = 6.59, *p* = 0.003, d = 1.06, posthoc power estimate = 0.99) (Fig. 6). Bonferroni-corrected post hoc comparisons revealed that deviations in GA > 4 mg/kg/h (–1.55 ± 0.93 mm, mean ± std, *n* = 11) were significantly larger compared to LA (–0.39 ± 0.91 mm, *n* = 22, *p* = 0.011). Furthermore, there was a significant difference in deviations of z-coordinates of the centers of final electrodes from the z-coordinate of the DBS sweetspot between the GA > 4 mg/kg/h vs GA ≤ 4 mg/kg/h groups ((–0.16 ± 1.22 mm, *n* = 17; *p* = 0.003). No significant difference was found between LA and GA ≤ 4 mg/kg/h (*p* = 0.99).

## Discussion

In this study, we assessed the graded effect of the sedative propofol on MER during DBS implantation surgery targeting the STN. We found that high propofol doses during MER increase the risk of misplacing the final DBS electrode. In contrasts, MER performed at propofol infusion rate ≤4 mg/kg/h yielded results comparable to those obtained under LA, both in terms of final lead placement and identification of electrophysiological hallmarks. High propofol doses predominantly suppressed neural activity in the dorsal part of the STN, which contains the clinical DBS sweetspot. Using objective sedation measures, we demonstrated a dose-dependent relationship with greater sedation depth corresponding to increased blurring of the dorsal STN border. BI, but not FR, indicated the imaging-defined ventral STN border under both LA and GA. Together, these findings provide practical guidance for intra-operative electrophysiologists performing DBS implantation surgery under GA.

MER serve as a complementary tool to preoperative imaging, aiding in the identification of the DBS target during electrode implantation surgery. Its value varies across cases - from simply confirming the preoperative imaging to providing decisive information that optimizes final electrode placement. Given the substantial advances in the availability of high-quality preoperative imaging, adjustments to the planned positioning of the electrode based on intra-operative MER are often subtle. Indeed, some studies have suggested that MER may become obsolete, as clinical outcomes and final electrode placements under GA without MER appear comparable at the group level to those achieved under LA with MER^5^. Similarly, 7 T preoperative imaging planning, currently available in only few centers, has further improved DBS targeting accuracy^16,17^. However, intra-operative brain shift and misalignment between preoperative imaging and intra-operative conditions can introduce unpredictable inaccuracies during DBS lead placement in individual surgeries^18^. Factors such as brain atrophy can increase brain shift risk^19^, yet the occurrence of brain shift cannot be predicted with accuracy and is influenced by factors including air inflow (pneumocephalus) and intra-operative loss of cerebrospinal fluid^20^. When brain shift occurs, MER become invaluable to avoide final electrode misplacement, which if it occurs can complicate clinical programming and compromise long-term therapeutic outcomes^15^. As more centers adopt DBS implantation surgery under GA, understanding how propofol alters the spatial and electrophysiological features of SUA becomes increasingly important. Previous studies have characterized propofol’s effect on STN neural activity, but group-level analyses in common anatomical space and direct comparisons with imaging-defined SNr are lacking. Clinically, it is essential to define propofol thresholds that should not be exceeded for MER to still reliably identify the DBS target under GA. When high doses of propofol are required, the intra-operative electrophysiologist must know which physiological hallmarks can still guide safe and accurate electrode placement in these challenging scenarios. To date, sufficiently powered studies addressing these clinically relevant questions are missing. The present study was designed to fill these gaps.

In MER performed under LA, we show in Fig. 2, and consistent with previous studies, that an increase in background noise marks the dorsal border of the STN. This reflects the transition from the fiber capsule dorsal to the STN to the cell-dense nucleus itself^2^. Under propofol-based GA, however, this physiological hallmark can be attenuated or lost, particularly at higher propofol infusion rates^21^. We replicate these observations at the group level and found that the NRMS signature of the STN is preserved when propofol is maintained below 4 mg/kg/h (Fig. 2). Accordingly, comparison of NRMS between LA and low-dose GA revealed no significant differences (Supplementary Fig. 3). One plausible explanation is that STN neurons may maintain characteristic neuronal firing patterns under low-dose propofol, possibly because they are physiologically accustomed to GABAergic modulation provided by afferents from the external part of the globus pallidus (GPe)^22^. Prior work has also suggested that STN spiking activity is more robust to GA than background unit activity, potentially allowing STN identification even when background unit activity is diminished or absent^21^. Indeed, defining the dorsal STN border relies on detecting characteristic neuronal activity with a distinct burst discharge pattern (Fig. 1E). Consistent with previous reports^21^ we found burst firing to be exaggerated under propofol-based GA (Figs. 1, 3 and 5). At the same time, high propofol levels have been shown to suppress neuronal spiking^13^ and our findings confirm that the occurrence of SUA is critically influenced by propofol dosage (Fig. 2). Importantly, corroborating prior work, we show that MER cannot accurately delineate the dorsal STN border when propofol infusion exceeds 4 mg/kg/h, resulting in missed targeting of the prescribed clinical DBS sweetspot^15^. To account for interindividual differences in propofol metabolism, we additionally assessed an objective metric of sedation depth derived from intra-operative EEG: the spectral edge frequency (SEF95). SEF95 correlated significantly with the accuracy of dorsal STN border identification, indicating that deeper sedation (lower SEF95) is associated with a more blurred STN entry (Fig. 4). When identification of the dorsal STN border is difficult or obscured due to high levels of propofol, distinguishing ventral STN border becomes particularly important, as it provides essential depth information along the z-axis of the trajectory. The ventral STN border is defined by the transition from the STN to the SNr, with the latter typically exhibiting less bursty and more high-frequency neuronal firing patterns^2^. However, evidence on SNr activity under GA is limited, and it remains unclear whether ventral STN border identification is feasible during propofol-based GA^4^.

In our study, we compared imaging-defined STN and SNr. Only BI, and not FR or CV, distinguished between these two structures, enabling ventral STN border discrimination under both LA and GA. When considering STN vs SNr as defined by the intra-operative electrophysiologist, we observed higher firing rates in the SNr than in the STN, consistent with previous reports^2^. However, because high firing rates and tonic firing patterns are criteria used for intra-operative differentiation of STN and SNr, comparisons based on these features can be considered circular and therefore limit interpretability. Our observation of no significant difference in FR between imaging-defined STN and SNr may reflect the cellular heterogeneity of the SNr, which contains both GABAergic and dopaminergic neurons that differ substantially in their firing rates; dopaminergic neurons exhibiting lower discharge frequencies than GABAergic neurons across species^23^. Despite this heterogeneity, BI differentiated imaging-defined STN from SNr, while FR did not (Fig. 3 and Supplementary Fig. 4). Regarding neuronal features (FR, BI, CoV) in our dataset, we did not find any significant correlations with clinical preoperative UPDRS III scores (Supplementary Fig. 2). This lack of correlative evidence is consistent with previous reports^24^ but contrasts with studies showing significant correlations between the subthalamic LFPs and clinical scores^25–27^. LFP measures may exhibit more robust correlations with clinical markers because they sample and integrate neural activity from a larger population of STN neurons, whereas single-unit recordings capture more fine-grained and spatially restricted aspects of neuronal activity^28^.

BI emerges as a potential identifier of the ventral STN boundary among the neuronal features evaluated. We show that, for STN neurons, BI is negatively correlated with SEF95, whereas FR shows a positive correlation (Fig. 4). Additionally, the distance of each MER site to the DBS sweetspot^15^ was significantly correlated with BI under both LA and GA conditions (Fig. 5), indicating that BI values tend to be higher when the respective MER site is closer to the DBS sweetspot. The spatial topography of BI observed in our dataset aligns with previous MER studies reporting a spatial affinity of tonic activity toward the ventral STN and burst activity towards the dorsal STN^29–31^. Our findings corroborate these prior observations, showing that the BI topography across the STN is preserved in GA, despite BI values being significantly higher and more variable under GA compared to LA (Figs. 2 and 5). Intra-operative detection of high BI may alert the neurophysiological team that MER are substantially influenced by the sedation level at the time of recording (Fig. 3). This can aid in interpreting potential mismatches between imaging- and electrophysiology-defined dorsal STN border (Fig. 4). In cases where sedation is carefully controlled, identifying the characteristic STN bursting pattern and a sudden increase in background unit activity remains crucial for accurately delineating the dorsal STN border (Fig. 2 and Supplementary Fig. 3). While our data suggest that BI is a useful spontaneously occurring biomarker to delineate STN and SNr and this diffrentiation is significant this differentiation for both LA and GA, the relatively small effect size (Cohen’s d = 0.27) suggests that consideration of additional mapping modalities may further increase certainty in assessment of the ventral STN border. Microstimulation, in particular, has proven valuable for confirming the ventral STN border. Specifically, prolonged inhibition of neuronal firing following low-amplitude, high-frequency microstimulation has been shown to be a consistent feature of SNr neurons and is rarely observed in STN neurons^32^. The distance to the internal capsule can also be estimated through intra-operative stimulation effects, and this assessment may be facilitated by concurrent EMG monitoring, a workflow often easier to implement under GA^33^. Ultimately, real-world GA-DBS workflows should integrate the occurrence and variability of MER features along electrode trajectories with intra-operative macro- and microstimulation findings. Considering the dose-dependent effects of propofol on the occurence of single-unit activity and recognizing that BI remains a potential neuronal biomarker under GA can substantially support the assessment of the ventral STN border.

Confirming previous findings, our results suggest that proximity to the DBS sweetspot is associated with postoperative clinical improvement (Supplementary Fig. 6), likely because optimally placed leads maximize the therapeutic window. Our data further show that DBS implantation surgery performed under deep sedation (propofol >4 mg/kg/h) carries a risk of adversely affecting final DBS lead placement (Fig. 6). In practice, it is generally sufficient to reduce the propofol dose below 4 mg/kg/h after the burr hole is made, on average approxunately 15 minutes prior to MER thereby preserving stable intra-operative conditions for MER mapping. Importantly, our finding that the clarity of the dorsal STN boundary scales with objective measures of sedation depth may help intra-operative teams interprete MER topologies when deep sedation is clinically required (Fig. 4). Under all other circumstances, our results support maintaining propofol infusion at ≤4 mg/kg/h. At or below this threshold, intra-operative electrophysiology can reliably identify hallmarks of spatial navigation (e.g., the dorsal STN border as indexed by NRMS increase and densely packed SUA) under GA, without inferiority compared to LA. Thus, our data suggest that the advantages of GA-based DBS implantation (e.g., improved patient comfort and reduced surgery time) can be achieved without compromising electrophysiology-guided spatial navigation and intra-operative DBS target identification.

In recent years, several anesthetic regimens have been considered for DBS implantation surgery^4,6,34,35^. While concerns have been raised regarding potential interactions between propofol and ketamine^36^, studies in neurosurgical patients have demonstrated that this combination can enhance cerebral oxygenation and hemodynamic stability compared to propofol alone^37^. Moreover, low-dose ketamine infusions have been shown to support high-quality microelectrode recordings comparable to awake conditions^38^. Thus, co-administration of low-dose ketamine may enable a reduction in propofol dosage, improving the conditions for electrophysiology-guided spatial navigation in DBS surgery. The feasibility of this approach for DBS surgery should be investigated explicitly in future studies.

The retrospective nature of this study introduces a potential risk of selection bias, as the choice to perform surgery under GA or LA was based on clinical indications rather than randomized allocation within a prospective design. In the present study, our goal was to capture a “real-world” representation of intra-operative electrophysiology acquired during DBS surgery. It is important to highlight that the clinical decision of the anesthetic regime during DBS surgery is driven by factors that are unlikely to determine neuronal features that are appreciated through MER. For example, a given patient might be frightened by the idea to be awake during surgery and thus might prefer the procedure to be performed under GA. This very individual preference is unlikely to influence MER. On the other hand, symptom severity (Supplementary Table 1) in itself does not drive the clinical decision regarding the anesthetic regime during DBS surgery^4^. For the given reasons, we believe it is unlikely that the clinical decision for LA vs. GA introduces substantial selection bias in our dataset. The tested propofol threshold of 4 mg/kg/h was chosen based on prior clinical experience and may be criticized as somewhat arbitrary. Nevertheless, our results suggest that this threshold provides a practical rule-of-thumb: both NMRS and SUA characteristics appear comparable under GA (≤4 mg/kg/h) and LA, enabling reliable electrophysiological delineation of the STN similar to awake surgeries (Fig. 2). Our recommendation to limit propofol infusion rates to ≤4 mg/kg/h during MER is therefore based on observed associations and does not represend a pharmacokinetically derived breakpoint. This threshold requires prospective validation and may not generalize across surgical centers, anesthetic workflows, or patient populations. Prospective studies designed to further substantiate these findings may be challenging to conduct, given that our data support maintaining propofol below 4 mg/kg/h whenever clinically feasible.

The findings presented in this study should inform, but not define, clinical practice. Prospective investigations are needed to validate propofol thresholds and confirm the robustness of MER-derived navigation markers under GA DBS conditions. Together, the data presented here show that propofol-based GA is feasible in DBS surgery, but it requires careful titration of dosage. Under LA, or under GA with propofol infusion maintained ≤4 mg/kg/h, the dorsal STN border can be accurately identified. In contrast, propofol doses >4 mg/kg/h increase the risk of missing the dorsal STN border and the clinical DBS sweetspot, potentially resulting to overly deep placements of final electrodes. In both LA and GA, BI distinguished imaging-defined STN from SNr and correlated with proximity to the clinical sweetspot. This suggests that BI may aid ventral STN border identification in GA, particularly when neuronal recordings can be “polluted” by high levels of propofol sedation. Ultimately, this study demonstrates that while single-unit activity topologies are altered under GA, they can still provide meaningful guidance for intra-operative navigation and target identification during DBS surgery.

## Methods

### Patient details

This study analyzed archival microelectrode recording data from 25 patients with PD who underwent DBS implantation surgery targeting the STN at Charité – Universitätsmedizin Berlin between June 2019 and September 2021. We included patients for whom routine intra-operative EEG data were available for retrospective analysis within a prospective study framework (clinicaltrials.gov - NCT03982953), enabling detailed examination of the relationship between objective measures of sedation depth and neural parameters. Of the 25 surgeries, 11 were performed under local anesthesia (LA, awake) and 14 under general anesthesia (GA) with varying levels of propofol and remifentanil (Supplementary Table 1). The decision to perform surgery under LA or GA was based entirely on clinical considerations/necessities, including factors such as anxiety, blood pressure stability, tremor severity, and anticipated surgery duration. Detailed clinical information is provided in Supplementary Table 1, and a flowchart of the inclusion process is shown in Fig. 1D. This retrospective study was conducted in accordance with the World Medical Association Declaration of Helsinki and was approved by the local Ethics Committee (EA4/024/52).

### Surgical procedure

The surgical procedure consisted of three stages: (1) skin incision and burr hole preparation; (2) MER and therapeutic window assessment; and (3) lead implantation and skin closure. Surgery was performed using the Leksell stereotactic frame (Elekta Instrument AB, Stockholm, Sweden). STN target coordinates were calculated as a composite from direct magnetic resonance imaging (T2-weighted axial sequences) and indirect atlas–based targeting with the Brainlab Elements stereotactic planning software (Brainlab SE, München, Germany). Surgery was performed off dopaminergic medication (>12 h after last medication). The implantation of the internal pulse generator was performed separately. During surgery, electrophysiological mapping was performed using 2–5 microelectrodes in an orthogonal (0°) or rotated (45°) Ben-Gun configuration, starting 10 mm above the calculated target (ventral STN border). When two microelectrodes were used, the second was advanced 2 mm posterolateral to the central (aimed at the calculated target) electrode trajectory. During the first and third stages of surgery, patients under GA received continuous propofol infusion to maintain deep sedation. After burr hole preparation, the propofol dose was reduced (Supplementary Table 1). Based on intra-operative assessments and preoperative anatomical planning, a microelectrode trajectory was selected, and its implantation depth was adjusted to optimize targeting of the clinical sweetspot (Fig. 1B, C).

### Data collection and postoperative analysis of electrophysiological data

Neurophysiological signals were acquired using the Neuro Omega navigation system (Alpha Omega Engineering, Ziporit, Israel). All neural signals were sampled at 44 kHz. MER were saved as segments at each recording depth and automatically allocated to the neurosurgical target distance. Segments contaminated by movement or stimulation artifacts were discarded. Recordings were excluded if they met the following quality criteria: (1) were shorter than 3 s (*n* = 33); (2) had a signal-to-noise <3 or displayed multi-unit activity in which single units could not be unequivocally isolated (*n* = 51); (3) with respect to imaging-based structure identification, recordings were excluded if the neurons’ anatomical locations were outside both the STN and the SNr (*n* = 28). These criteria follow established methodological standards in MER studies^24,39,40^ and were applied uniformly across anesthesia conditions, making systematic bias unlikely. The final dataset included 583 high-quality single-unit recordings (mean signal-to-noise ratio 8.01 ± 3.02) from 25 patients (50 hemispheres, 128 trajectories). For analysis of single unit activity, we used a recently developed Python-based graphical user interface (https://github.com/Toronto-TNBS/spooky-spikes) that assesses firing rate as the inverse of the mean interspike interval (ISI) distribution, burst index as the ratio of means from a two-component Gaussian mixture model applied to the log ISI distribution, and the coefficient of variation by dividing the median absolute deviation of the ISI distribution by its median. Normalized root mean square (NRMS) was computed as the raw multi-unit activity recorded by the microelectrode at each electrode depth as previously described^40^. To achieve normalization, the NRMS values along the trajectory were scaled by dividing them by the median of the first 5 recording sites within that trajectory, each with a minimum duration of 10 s.

### Anesthetics and intra-operative EEG

Doses of sedatives and anesthetics (propofol and remifentanil) administered during electrode implantation were extracted from clinical records (Supplementary Table 1). Depth of sedation was assessed using frontal EEG channels (SedLine®, Masimo, Irvine, USA) recorded from four forehead electrodes (Fig. 1A). EEG data analysis was performed using the EEGLAB toolbox within the MATLAB environment, as previously reported^41,42^ toolbox available at https://github.com/mahtamsv/intraopEEGtoolbox). Data from every channel were concatenated across multiple data files available for that participant, epoched in non-overlapping 2-s windows. Epochs contaminated with artifacts were removed. Next, posthoc power estimate spectral density (PSD) was calculated for the pre-processed data for each participant as normalized PSD averaged across epochs and channels. Spectral edge frequency at 95% (SEF95) was then estimated as the frequency below which 95% of the total EEG posthoc power estimate is contained serving as a compact and robust metric for quantifying the depth of sedation^43^. The time interval of MER was identified by temporal alignment of the test stimulation (performed subsequently to the MER) artifact, that was visible from the raw EEG trace. The SEF95 for the respective time window of MER was considered for further analysis.

### Imaging—procedure of localizing MER to MNI space

Pre- and postoperative imaging were co-registered and normalized using the default pipeline as implemented in Lead-DBS v3.0^44^ yielding reconstruction of the trajectory of the final electrode in MNI space. DBS electrodes were subsequently reconstructed using the phantom-validated Precise and Convenient Electrode Reconstruction for Deep Brain Stimulation (PaCER) algorithm and manually refined, if necessary. Intra-operative recording sites were estimated by projecting back along this trajectory, accounting for potential intra-operative adjustments of implantation depth (Fig. 1F). This enabled mapping of SUA and NRMS across electrode sites in MNI space. MER localization considered postoperative imaging to avoid possible localization errors due to intra-operative brain shift. To determine the location of each MER relative to the anatomical substrates we used the DISTAL atlas as implemented in Lead-DBS^45^.

### Statistics

To align trajectory depths across recordings (Fig. 2), we defined a common reference midpoint based on the average entry and exit depths of the STN. The imaging-defined STN length for each trajectory was determined by extracting its entry and exit points. To establish a standardized reference, we calculated the midpoint of the mean imaging-defined STN lengths across all trajectories (2.24 mm) and adjusted individual trajectory depths by shifting them according to the difference between their individual and reference midpoints. Fourteen trajectories that did not reach the imaging-defined STN but reached the SNr, and six trajectories with an STN entry-to-exit length shorter than 3 mm, were excluded from the analysis. Based on prior intra-operative clinical experience, we grouped NRMS and SUA as follows: LA group with no propofol (trajectories 1–49), GA with propofol >4 mg/kg/h (trajectories 50–77), and GA with propofol ≤4 mg/kg/h (trajectories 78–108). NRMS was compared at the group level (Fig. 2B) using a cluster-based paired-permutation test^46^ to identify regions of significant NRMS differences between conditions at aligned recording depths. To correct for multiple comparisons in NMRS analysis across recording depths, a Benjamini–Hochberg false discovery rate (FDR) correction was applied. On Fig. 2D the depth of isolated SUA was compared using a one-way ANOVA (1×3), with post hoc t-tests corrected for multiple comparisons (Bonferroni, *n* = 3). Furthermore, for Fig. 2E we assessed between-group spatial differences in MNI-coordinates on the XYZ dimensions using multivariate ANOVA (MANOVA) with post-hoc t-test and Bonferroni correction. A vector machine (SVM) algorithm was trained to discriminate between anesthesia groups using the spatial location of recording sites in MNI space as input features. In order to assess stability of the SVM algorithm, we permuted the data 1000x and repeated the classification (ROC of the groups plotted as faint curves). (Supplementary Fig. 1). Neuronal features (FR, BI and CV) were analyzed using a two-way ANOVA (2×2) with factors “structure” (STN vs. SNr) and “anesthesia” (LA vs. GA). Comparisons were performed for STN and SNr as defined by fusing pre- and postoperative imaging (Fig. 3). Additional comparisons using intra-operative team-defined STN and SNr are provided in Supplementary Fig. 5. Post hoc t-tests were Bonferroni-corrected for multiple comparisons (*n* = 4). The relationship between an objective measure of the depth of sedation and the accuracy of dorsal STN border differentiation, as well as neuronal features, was assessed using Spearman correlations (Bonferroni corrected, *n* = 4) between SEF95 and the normalized distance of the first SUA from the dorsal STN border and neuronal features (FR, BI, and CoV) (Fig. 4). Note for the analysis of dorsal STN border differentiation, we excluded SUA that did not present itself in proximity to a minimum of two other isolated single units along the trajectory to minimize accidental inclusion of the zona incerta (ZI) neurons, as the ZI contains scarcely spaced neurons and is thus distinct from densely spaced STN neurons. We also excluded trajectories that did not transverse the imaging-defined STN for at least 3 mm. Separate Spearman correlations (Bonferroni corrected, *n* = 2) assessed the relationship between the distance MER sites to the clinical DBS sweetspot^15^ with the burst index of a respective MER site (Fig. 5). Final electrode placement was evaluated based on MNI coordinates of the center of each electrode, defined as the midpoint of all contact levels (Fig. 6). One-way ANOVA (1×3) was used to compare Euclidean distance to the DBS sweetspot and the z-coordinate of the center of the final DBS lead across groups (LA, propofol >4 mg/kg/h, propofol ≤4 mg/kg/h). Post hoc t-tests were Bonferroni corrected (*n* = 3).

## Supplementary information

supplementary_revised_npjparkd

## Data Availability

The datasets analyzed during the current study is available from the corresponding author upon reasonable request.

## Code availability

All code used in this study is available in the Toronto-TNBS/spooky-spikes repository https://github.com/Toronto-TNBS/spooky-spikes and for intraoperative EEG analyses https://github.com/mahtamsv/intraopEEGtoolbox and https://www.lead-dbs.org/download/.
