# Supplementary material for: The graded effect of propofol in electrophysiology-guided navigation during deep brain stimulation surgery
Source: NPJ Parkinsons Dis. 2026 Jan 29;12:64. doi: 10.1038/s41531-025-01243-1 (PMC12992798; doi:10.1038/s41531-025-01243-1)
Supplement: Supplementary file 1 — supplementary_revised_npjparkd [file 41531_2025_1243_MOESM1_ESM.pdf]

# Supplementary material

## Table of Contents

Supplementary Table 1: Demographics and intraoperative information of patients

Supplementary Figure 1: Overview of microelectrode trajectories mapped single-unit recording sites, and spatial clustering of MNI coordinates across anesthesia conditions

Supplementary Figure 2. Correlation between neuronal features and global motor severity (MDS-UPDRS-III OFF scores) under local anesthesia

Supplementary Figure 3. Cluster-based permutation test on normalized root mean square (NRMS) activity across anesthesia conditions.

Supplementary Figure 4: Neuronal features in imaging-defined STN and SNr under general anesthesia separated in propofol high-dose (> 4 mg/kg/h) and low-dose (≤ 4 mg/kg/h) groups

Supplementary Figure 5: Comparison of neuronal features between intraoperatively electrophysiology-defined STN vs SNr under local and general anesthesia

Supplementary Figure 6. Relationship between electrode center and proximity to DBS sweetspot with therapeutic outcome

Supplementary Figure 7. Relationship between propofol dosage (mg/kg/h) vs and intraoperative EEG spectral edge frequency (SEF95)

**Supplementary Table 1**

Demographics and intraoperative information of patients.

| Anesthetic technique | Patient number | Age year/ gender | Clinical subtype | Most affected side | Disease duration (years) | UPDRS III off levodopa | UPDRS III on levodopa | H&Y staging | Levodopa equivalent dose | First surgery side | Number of trajectory (L/R) | Number of sorted single neurons | Average recording length (sec) | IntraSTN traj. length (L/R; mm) | Reduced propofol (L/R; mg/kg/h) | Reduced remifentanil (L/R; mg/kg/h) | Level of sedation (L/R; SEF 95%) |
|---|---|---|---|---|---|---|---|---|---|---|---|---|---|---|---|---|---|
| LA | 1 | 56/F | Equivalent | R | 13 | 102 | 28 | IV | 1015 | R/L | 2/3 | 31 | 10.8 | 5.5/6.4 | - / - | - / - | 25.0/25.0 |
| | 2 | 44/F | Equivalent | L | 13 | 48 | 29 | II | 1020 | R/L | 4/4 | 39 | 14.8 | 7.0/6.2 | - / - | - / - | 23.5/20.5 |
| | 3 | 53/M | Equivalent | L | 08 | 59 | 7 | II | 1114 | L/R | 1/1 | 07 | 14.2 | 6.8/5.7 | - / - | 0.05/0.03 | 24.0/21.5 |
| | 4 | 59/M | Akinetic-rigid | R | 06 | 54 | 14 | II | 1597 | L/R | 3/4 | 34 | 10.9 | 6.7/6.4 | - / - | - / - | 22.5/23.5 |
| | 5 | 61/F | Akinetic-rigid | L | 05 | 50 | 33 | II | 598.5 | R/L | 3/3 | 11 | 11.7 | 5.4/6.2 | - / - | - / - | 25.5/22.5 |
| | 6 | 72/M | Akinetic-rigid | L | 15 | 67 | 43 | III | 734 | L/R | 2/2 | 27 | 11.4 | 6.2/6.3 | - / - | 0.05/0.05 | 22.0/25.5 |
| | 7 | 54/M | Akinetic-rigid | R | 04 | 69 | 32 | III | 1187 | L/R | 2/3 | 33 | 19.4 | 6.5/5.5 | - / - | - / - | 24.0/25.5 |
| | 8 | 62/M | Akinetic-rigid | R | 07 | 26 | 11 | III | 1048 | L/R | 5/2 | 17 | 14.8 | 3.5/4.7 | - / - | 0.03/0.03 | 21.5/23.0 |
| | 9 | 55/M | Akinetic-rigid | L | 07 | 25 | 5 | II | 1596 | R/L | 2/2 | 26 | 5.20 | 5.6/6.3 | - / - | 0.03/0.03 | 22.0/20.0 |
| | 10 | 54/F | Tremor-dominant | R | 09 | 55 | 13 | II | 1266 | L/R | 3/2 | 29 | 5.30 | 5.9/6.2 | - / - | - / - | 19.5/15.0 |
| | 11 | 69/F | Akinetic-rigid | R | 11 | 76 | 43 | IV | 1299 | R/L | 3/2 | 21 | 5.40 | 5.3/6.0 | - / - | 0.40/0.30 | 15.0/15.0 |
| TOTAL | 11 | 58±7; 6/5 | | | 8±3 | 57±21 | 23±14 | | 1134 | 22 | 30/28 | 285 | 11.2 | | | | |
| GA | 1 | 57/M | Tremor-dominant | L | 02 | 83 | 57 | II | 1166 | R/L | 2/2 | 13 | 16.0 | 3.2/5.1 | 3.0/3.0 | 0.15/0.15 | 14.0/14.0 |
| | 2 | 66/M | Akinetic-rigid | R | 08 | 63 | 30 | II | 1250 | R/L | 1/2 | 08 | 19.5 | 5.6/3.0 | 6.0/6.0 | 0.50/0.30 | 14.5/14.5 |
| | 3 | 69/M | Akinetic-rigid | L | 08 | 57 | 23 | II | 1449 | L/R | 3/2 | 15 | 10.8 | 5.3/6.1 | 1.0/1.0 | 0.05/0.003 | 13.0/15.0 |
| | 4 | 69/F | Tremor-dominant | L | 10 | 49 | 30 | III | 1451 | L/R | 3/3 | 28 | 14.8 | 6.6/5.7 | 7.5/7.5 | 0.20/0.40 | 16.5/16.5 |
| | 5 | 57/F | Tremor-dominant | R | 16 | 42 | 22 | III | 1413 | R/L | 2/2 | 13 | 20.3 | 5.1/6.5 | 5.5/5.0 | 0.20/0.30 | 15.3/14.0 |
| | 6 | 64/F | Akinetic-rigid | L | 16 | 56 | 35 | IV | 1098 | R/L | 2/3 | 43 | 11.2 | 6.0/7.1 | 3.5/3.5 | 0.15/0.15 | 14.0/17.5 |
| | 7 | 62/M | Akinetic-rigid | R | 11 | 44 | 26 | IV | 1131 | R/L | 2/2 | 16 | 16.5 | 6.2/5.7 | 3.5/5.0 | 0.20/0.20 | 11.5/11.0 |
| | 8 | 68/M | Akinetic-rigid | R | 17 | 40 | 25 | III | 1373 | R/L | 2/3 | 08 | 14.4 | 2.3/2.8 | 4.0/4.0 | 0.20/0.40 | 12.0/12.0 |
| | 9 | 57/F | Akinetic-rigid | R | 16 | 57 | 6 | III | 908 | L/R | 3/2 | 32 | 17.1 | 5.7/6.1 | 2.5/2.5 | 0.30/0.20 | 11.5/12.5 |
| | 10 | 61/M | Tremor-dominant | R | 11 | 37 | 23 | III | 1064 | R/L | 3/3 | 24 | 16.3 | 6.4/6.1 | 2.0/2.0 | 0.10/0.10 | 13.5/13.5 |
| | 11 | 60/F | Akinetic-rigid | R | 03 | 50 | 9 | II | 1083 | L/R | 4/5 | 48 | 15.7 | 5.8/6.1 | 1.5/1.5 | 0.02/0.03 | 23.0/23.5 |
| | 12 | 71/M | Akinetic-rigid | R | 06 | 43 | 25 | III | 1080 | R/L | 2/2 | 11 | 13.5 | 6.7/5.9 | 5.0/5.5 | 0.30/0.30 | 15.5/15.0 |
| | 13 | 60/M | Akinetic-rigid | L | 11 | 49 | 35 | III | 1347 | R/L | 2/4 | 23 | 13.5 | 3.3/5.5 | 4.0/5.0 | 0.20/0.20 | 18.5/17.5 |
| | 14 | 62/F | Tremor-dominant | R | 06 | 55 | 38 | III | 1074 | R/L | 2/2 | 16 | 15.1 | 6.8/5.4 | 4.5/4.5 | 0.25/0.20 | 18.5/18.5 |
| TOTAL | 14 | 63±5; 8/6 | | | 10±4 | 52±12 | 27±12 | | 1206 | 28 | 33/37 | 298 | 15.3 | | | | |

UPDRS, unified Parkinson's disease rating scale; H&Y, Hoehn & Yahr stage; L, left; R, right

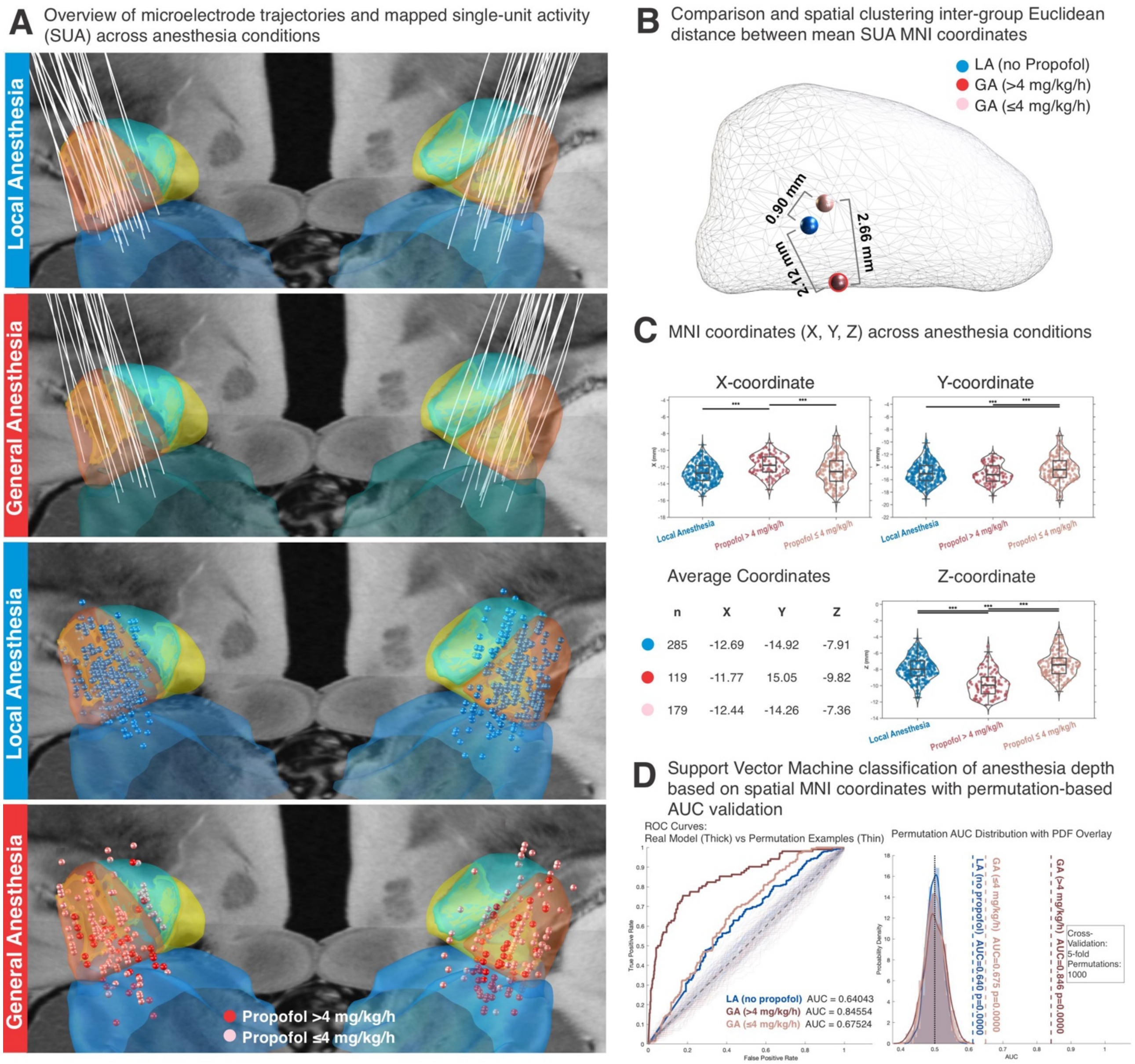


**Supplementary Fig. 1 | Overview of microelectrode trajectories mapped single-unit recording sites, and spatial clustering of MNI coordinates across anesthesia conditions. A** Posterior view of microelectrode trajectories and corresponding SUA in the STN and SNr across 25 patients with Parkinson's disease undergoing STN-DBS surgery. A total of 128 trajectories and 583 SUA recordings are shown in MNI space. Top two panels: Corresponding MER trajectories for the same patients in LA and GA. Bottom two panels: Mapped SUA locations from surgeries performed under local anesthesia (LA, blue dots; n = 285 SUA from 11 patients, 58 trajectories) and general anesthesia (GA, red (propofol >4 mg/kg/h, n = 119) and pink dots (propofol ≤4 mg/kg/h, n = 179); n = 298 SUA from 14 patients, 70 trajectories). **B** Average SUA spatial locations depicted in MNI space for each group with inter-group Euclidean distances to highlight proximity of the spatial distribution. **C** The MANOVA revealed significant inter-group differences in the SUA XYZ coordinates (Wilks' $\Lambda = 0.71$, $F_{6,569} = 17.57$, $p < .001$, $d = 0.86$, posthoc posthoc power estimate > 0.99). Post-hoc paired comparison tests

demonstrated a specific pattern of inter-group differences in SUA spatial distribution ($p < 0.001$), mainly in the z-axis. Significance was Bonferroni-corrected for multiple comparison. **D** As an exploratory validation, a Support Vector Machine (SVM) model achieved fair-to-good discrimination across all conditions (5-kfolds), with an average AUC of 0.71. The algorithm performance in discriminating each group is plotted as colored Receiver Operating Characteristics (ROC) curves in the left panel with AUC of 0.64 for no propofol (LA) group is 0.67 for Propofol ≤4 mg/kg/h group, and 0.85 for >4 mg/kg/h group. SVM stability was confirmed by 1000× permutation (faint ROC curves), with performance preserved as shown in the right-panel density plot.

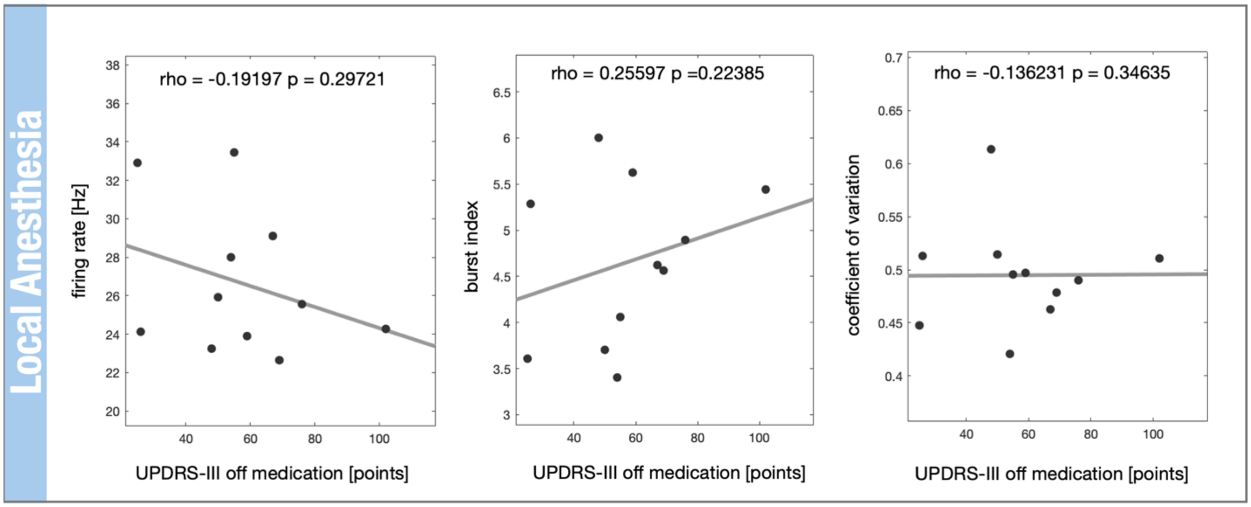


**Supplementary Fig. 2 | Correlation between neuronal features under local anesthesia and global motor impairment severity (MDS-UPDRS-III OFF scores).** Each dot represents the patient-specific median value of neuronal features: firing rate (FR), burst index (BI), and coefficient of variation (CoV) plotted against the corresponding total MDS-UPDRS-III OFF score. R- and p-values are taken from separate Spearman correlations. Gray lines represent best-fit linear regression trends.

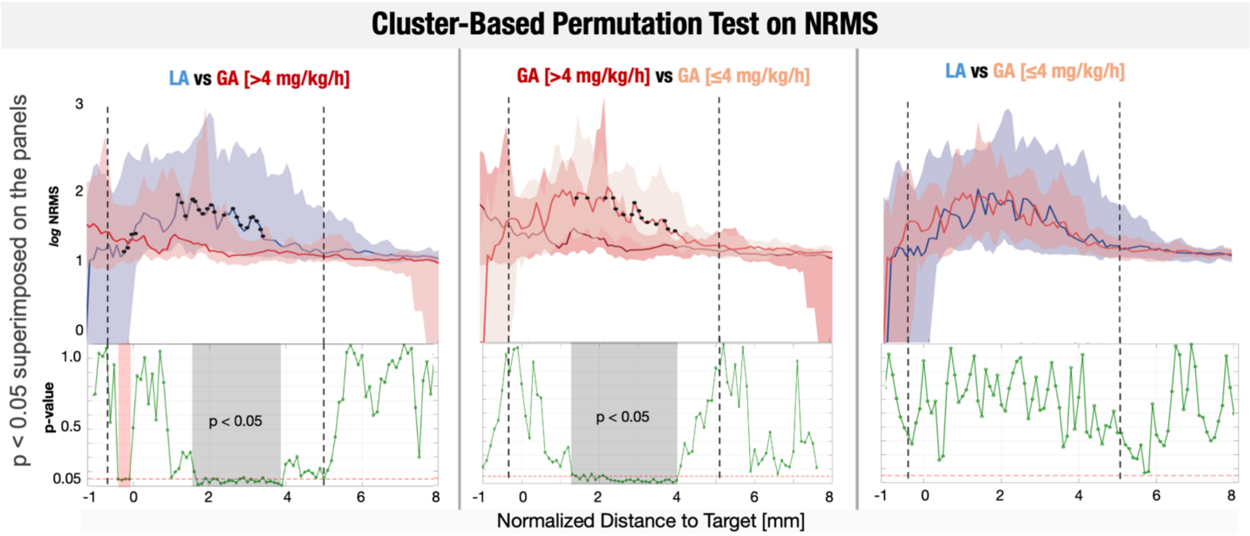


**Supplementary Fig. 3 | Cluster-based permutation test on normalized root mean square (NRMS) activity across anesthesia conditions.** Group-level comparisons of NRMS were performed between local anesthesia (LA, blue), high-dose general anesthesia (GA > 4 mg/kg/h, dark red), and low-dose general anesthesia (GA ≤ 4 mg/kg/h, light red). The shaded areas represent the mean ± SEM of NRMS values along the normalized depth axis relative to the surgical target. Black dots denote locations with significant $p < 0.05$ differences, superimposed on the NRMS profiles, while the lower panels display the corresponding permutation-based p-value distributions. A significant cluster was observed for LA vs. GA (> 4 mg/kg/h) between 3.6 mm and 1.2 mm above the target (grey shaded zone; $p < 0.05$, FDR-corrected), indicating higher NRMS values under LA. A second significant cluster was identified for GA (> 4 mg/kg/h) vs. GA (≤ 4 mg/kg/h) between 3.9 mm and 1.5 mm above target (grey shaded zone; $p < 0.05$, FDR-corrected), with higher NRMS values under low-dose GA. In contrast, no significant cluster emerged for LA vs. GA (≤ 4 mg/kg/h) comparisons, suggesting that low-dose propofol preserves NRMS activity patterns comparable to those observed under LA.

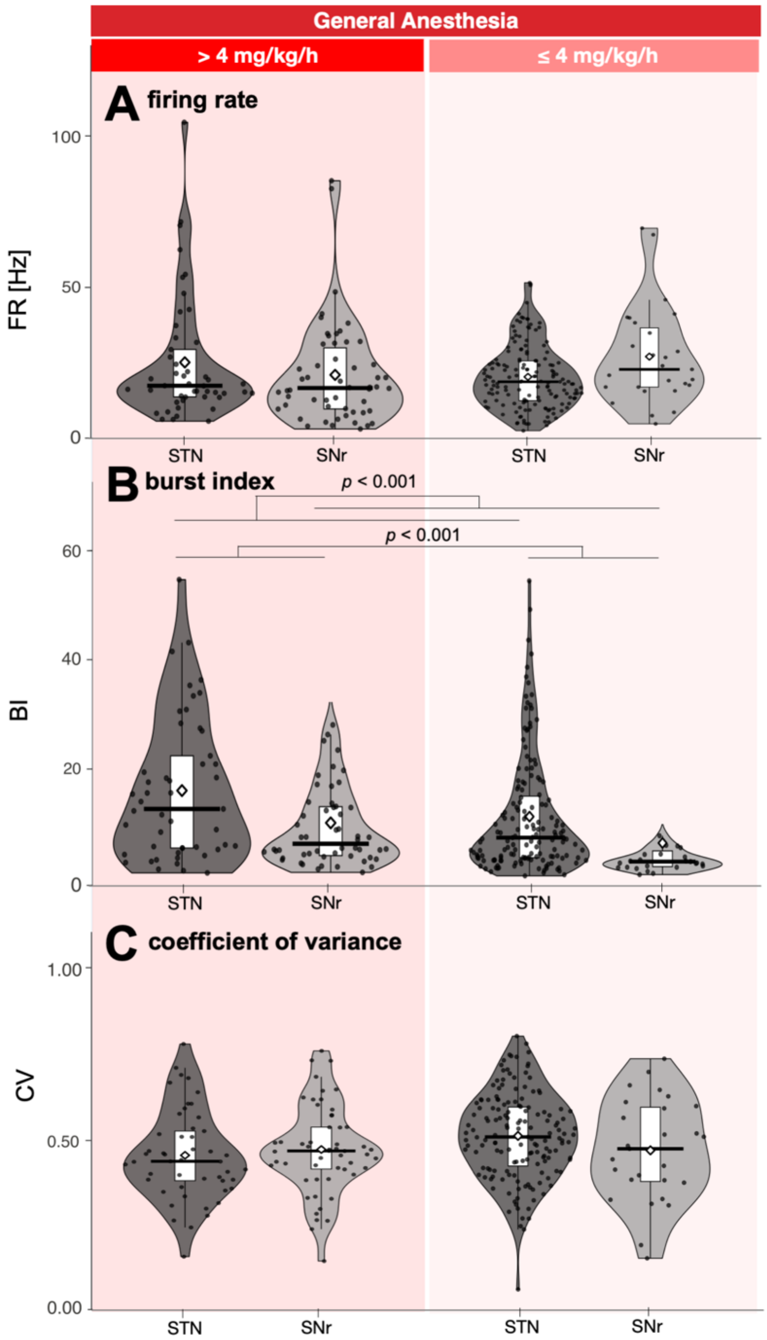


**Supplementary Fig. 4 | Comparison of neuronal features in imaging-defined STN and SNr under general anesthesia in propofol high-dose (> 4 mg/kg/h) and low-dose (≤ 4 mg/kg/h) groups. A** Analysis of variance of firing rate for imaging defined STN revealed no significant main effects of structure ($F_{1,281}$ = 1.05, p = 0.305, d =0.12; posthoc power estimate = 0.17) or anesthesia ($F_{1,281}$ = 1.70, p = 0.193, d = 0.16; posthoc power estimate = 0.26), and no significant structure × anesthesia interaction ($F_{1,281}$ = 6.77, p = 0.109, d = 0.31; posthoc power estimate = 0.76). **B** For the burst index, a significant main effect was found for anesthesia ($F_{1,281}$ = 8.41, p = 0.004, d = 0.35; posthoc power estimate = 0.83) and for structure ($F_{1,281}$ = 7.25, p = 0.007, d = 0.32; posthoc power estimate = 0.76), with no significant interaction ($F_{1,281}$ = 0.18, p = 0.672, d = 0.05; posthoc power estimate = 0.07). The anesthesia effect reflected higher BI values in the high-dose GA group (16.94 ± 1.52, mean ± SD; n = 281) compared to the low-dose GA group (7.52 ± 2.05; n = 281). The structure effect was driven by

higher BI values in the STN (12.37 ± 0.85; n = 281) compared to the SNr (7.52 ± 2.05; n = 281). **C** Analysis of variance for the coefficient of variation (CV) revealed no significant main effects of structure ($F_{1,281}$ = 3.140, p = 0.0775, d = 0.21; posthoc power estimate = 0.44), condition ($F_{1,281}$ = 6.687, p = 0.0502, d = 0.31; posthoc power estimate = 0.82), or structure × condition interaction ($F_{1,281}$ = 2.512, p = 0.1141, d = 0.19; posthoc power estimate = 0.36).

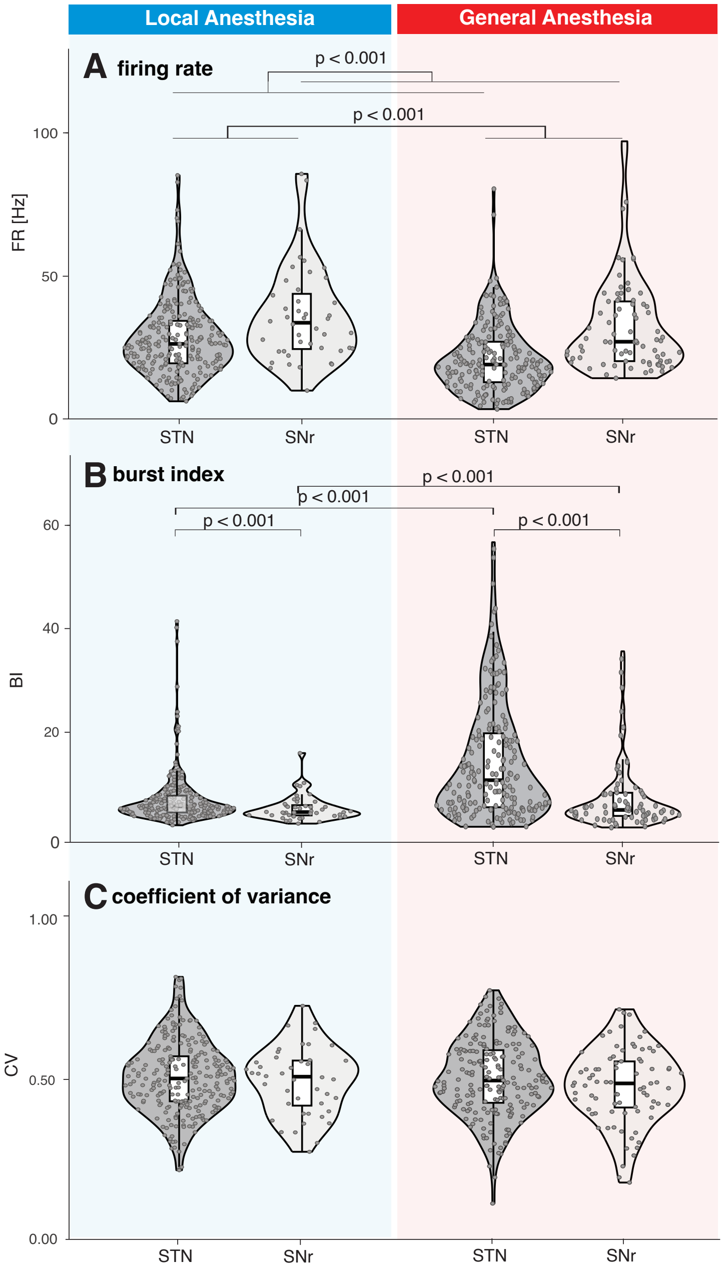


**Supplementary Fig. 5 | Comparison of neuronal features between intraoperatively electrophysiology-defined STN vs SNr under local and general anesthesia**. **A** Analysis of variance for firing rate for electrophysiology-defined STN revealed a significant main effect for anesthesia ($F_{1,579}$ = 40.330, *p* < 0.001, d = 0.53, posthoc power estimate = 0.99) and main effect of structure ($F_{1,579}$ = 44.650, *p* < 0.001, d = 0.56, posthoc power estimate = 0.99) but not on structure*anesthesia interaction ($F_{1,579}$ = 0.798, p = 0.372, d = 0.07, posthoc power estimate = 0.15).

The main effect of anesthesia was driven by higher firing rates in the LA group (31.46 ± 14.01 Hz, mean ± SD; n = 301) compared to the GA group (22.86 ± 15.29 Hz; n = 282). The main effect of structure was due to higher firing rates in the SNr (31.69 ± 13.53 Hz; n = 125) compared to the STN (22.63 ± 13.01 Hz; n = 458). **B** Analysis of variance for burst index for imaging defined STN revealed a significant main effect for anesthesia ($F_{1,579}$ = 48.0469, p < 0.0001, d = 0.58, posthoc power estimate = 0.99), a significant main effect of structure ($F_{1,579}$ = 22.127, p < 0.0001, d = 0.39, posthoc power estimate = 0.99), and a significant interaction between structure and anesthesia ($F_{1,579}$ = 7.837, p = 0.005, d = 0.23, posthoc power estimate = 0.80). Pairwise comparisons revealed a significant difference in burst index under general anesthesia (STN_GA = 14.22 ± 7.92, mean ± SD; n = 201 vs. SNr_GA = 7.99 ± 7.93; n = 81; p < 0.0001), but not under local anesthesia (STN_LA = 6.14 ± 7.90; n = 257 vs. SNr_LA = 4.56 ± 7.91; n = 44; p = 0.222). **C** Analysis of variance for the coefficient of variation (CV) revealed no significant main effects of structure ($F_{1,579}$ = 2.51, p = 0.114, d = 0.13, posthoc power estimate = 0.33), condition ($F_{1,579}$ = 1.63, p = 0.686, d = 0.11, posthoc power estimate = 0.24), or structure × condition interaction ($F_{1,579}$ = 0.324, p = 0.569, d = 0.05, posthoc power estimate = 0.09).

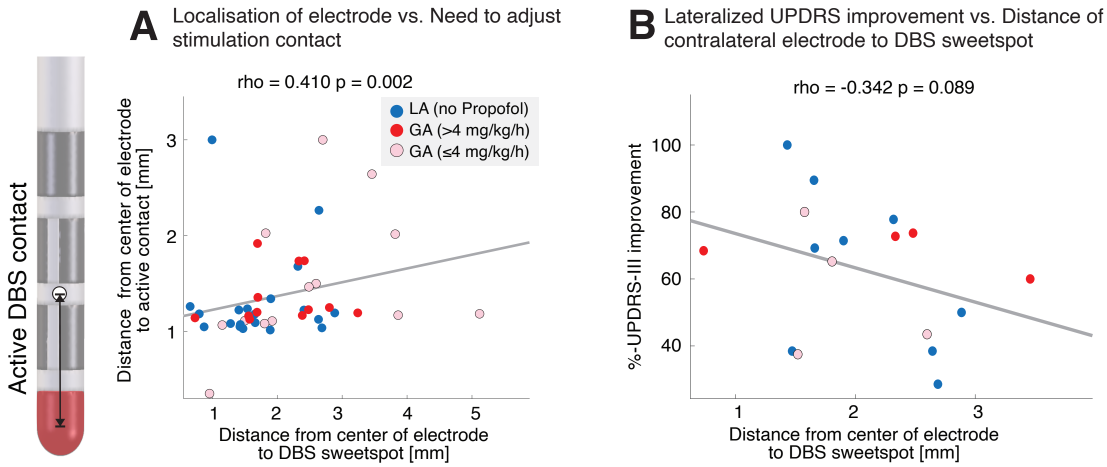


**Supplementary Fig. 6 | Relationship between electrode center and active contact and proximity of center to DBS sweetspot with therapeutic outcome. A** Scatter plot showing the relationship between the distance of the active DBS contact from the electrode center and the distance of the center of the electrode to the clinical DBS sweetspot (Dembeck et al. 2019). The positive Spearman correlation (n = 44, ρ = 0.420, p = 0.002; d = 0.93, posthoc power estimate = 0.82) suggests that more sophisticated programming is necessary if the center of electrode is further away from the DBS sweetspot. **B** Scatter plot illustrating the relationship between the therapeutic effect of the more affected side (lateralized percentage UPDRS-III improvement) and the distance of the active DBS contact from the electrode center to the DBS sweetspot on the contralateral side. The trend towards a significant negative correlation (n = 17, rho = -0.342, p = 0.089, d = -0.73, posthoc power estimate

= 0.27) indicates that closer proximity to the sweetspot is associated with greater clinical improvement. Data points are color-coded by anesthesia condition: light blue = LA (no propofol), red = GA (> 4 mg/kg/h), and dark blue = GA (< 4 mg/kg/h). Spearman's rho values are reported in each panel along with corresponding p-values.

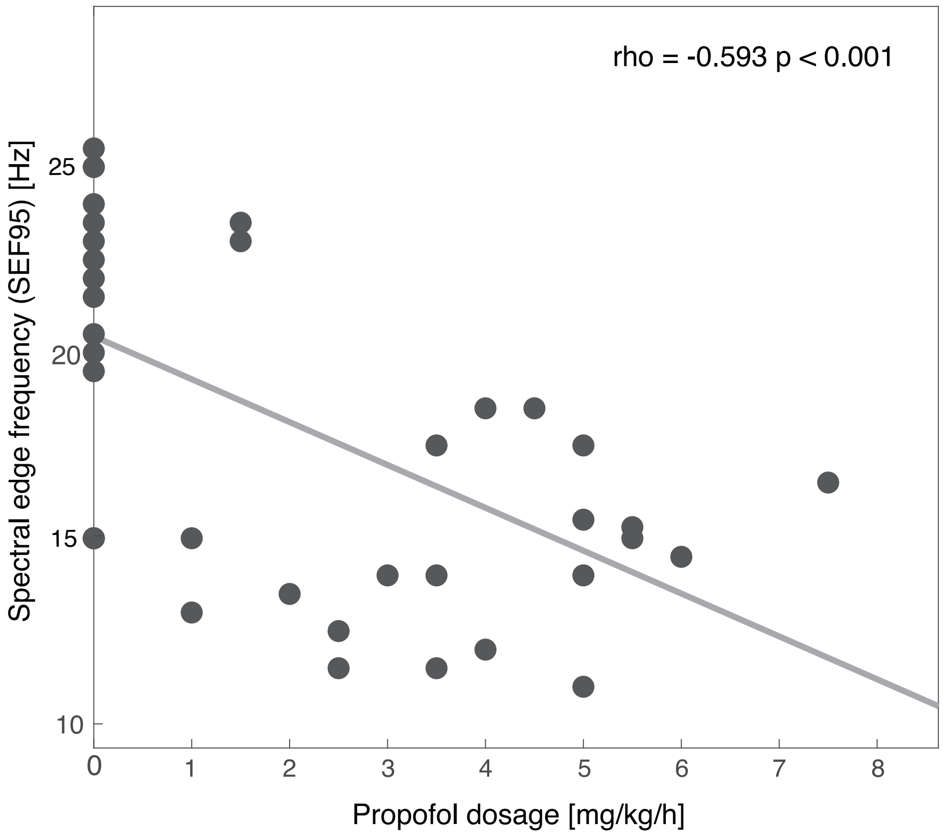


**Supplementary Fig. 7 | Relationship between propofol dosage (mg/kg/h) and intraoperative EEG spectral edge frequency (SEF95).** Each dot represents a single hemisphere recording. The x-axis depicts the propofol infusion dose during microelectrode recordings, while patients operated under local anesthesia (LA) are included with a dosage of 0 mg/kg/h. A significant negative correlation ($n = 50$, $\rho = -0.593$, $p < 0.001$, $d = –1.47$, posthoc power estimate = 0.997) was observed, indicating that higher propofol doses were associated with lower SEF95 values, consistent with deeper levels of sedation.

| Section/topic | No | CONSORT 2025 checklist item description | Reported on page no. |
|---|---|---|---|
| **Title and abstract** | | | |
| Title and structured abstract | 1a | Identification as a randomised trial | |
| | 1b | Structured summary of the trial design, methods, results, and conclusions | 2 |
| **Open science** | | | |
| Trial registration | 2 | Name of trial registry, identifying number (with URL) and date of registration | |
| Protocol and statistical analysis plan | 3 | Where the trial protocol and statistical analysis plan can be accessed | |
| Data sharing | 4 | Where and how the individual de-identified participant data (including data dictionary), statistical code and any other materials can be accessed | 19 |
| Funding and conflicts of interest | 5a | Sources of funding and other support (eg, supply of drugs), and role of funders in the design, conduct, analysis and reporting of the trial | 19 |
| | 5b | Financial and other conflicts of interest of the manuscript authors | 19 |
| **Introduction** | | | |
| Background and rationale | 6 | Scientific background and rationale | 3-4 |
| Objectives | 7 | Specific objectives related to benefits and harms | 4 |
| **Methods** | | | |
| Patient and public involvement | 8 | Details of patient or public involvement in the design, conduct and reporting of the trial | 14 |
| Trial design | 9 | Description of trial design including type of trial (eg, parallel group, crossover), allocation ratio, and framework (eg, superiority, equivalence, non-inferiority, exploratory) | 14 |
| Changes to trial protocol | 10 | Important changes to the trial after it commenced including any outcomes or analyses that were not prespecified, with reason | |
| Trial setting | 11 | Settings (eg, community, hospital) and locations (eg, countries, sites) where the trial was conducted | 14 |
| Eligibility criteria | 12a | Eligibility criteria for participants | 14 |
| | 12b | If applicable, eligibility criteria for sites and for individuals delivering the interventions (eg, surgeons, physiotherapists) | |
| Intervention and comparator | 13 | Intervention and comparator with sufficient details to allow replication. If relevant, where additional materials describing the intervention and comparator (eg, intervention manual) can be accessed | 14-15 |
| Outcomes | 14 | Prespecified primary and secondary outcomes, including the specific measurement variable (eg, systolic blood pressure), analysis metric (eg, change from baseline, final value, time to event), method of aggregation (eg, median, proportion), and time point for each outcome | 14-17 |
| Harms | 15 | How harms were defined and assessed (eg, systematically, non-systematically) | |
| Sample size | 16a | How sample size was determined, including all assumptions supporting the sample size calculation | 17 |
| | 16b | Explanation of any interim analyses and stopping guidelines | |
| Randomisation: | | | |
| Sequence generation | 17a | Who generated the random allocation sequence and the method used | 15 |
| | 17b | Type of randomisation and details of any restriction (eg, stratification, blocking and block size) | |

| | | | Reported on page no. |
|---|---|---|---|
| Allocation concealment mechanism | 18 | Mechanism used to implement the random allocation sequence (eg, central computer/telephone; sequentially numbered, opaque, sealed containers), describing any steps to conceal the sequence until interventions were assigned | |
| Implementation | 19 | Whether the personnel who enrolled and those who assigned participants to the interventions had access to the random allocation sequence | |
| Blinding | 20a | Who was blinded after assignment to interventions (eg, participants, care providers, outcome assessors, data analysts) | |
| | 20b | If blinded, how blinding was achieved and description of the similarity of interventions | |
| Statistical methods | 21a | Statistical methods used to compare groups for primary and secondary outcomes, including harms | 17 - 18 |
| | 21b | Definition of who is included in each analysis (eg, all randomised participants), and in which group | |
| | 21c | How missing data were handled in the analysis | |
| | 21d | Methods for any additional analyses (eg, subgroup and sensitivity analyses), distinguishing prespecified from post hoc | 17 |
| **Results** | | | |
| Participant flow, including flow diagram | 22a | For each group, the numbers of participants who were randomly assigned, received intended intervention, and were analysed for the primary outcome | 4 |
| | 22b | For each group, losses and exclusions after randomisation, together with reasons | |
| Recruitment | 23a | Dates defining the periods of recruitment and follow-up for outcomes of benefits and harms | |
| | 23b | If relevant, why the trial ended or was stopped | |
| Intervention and comparator delivery | 24a | Intervention and comparator as they were actually administered (eg, where appropriate, who delivered the intervention/comparator, how participants adhered, whether they were delivered as intended (fidelity)) | 4 - 6 |
| | 24b | Concomitant care received during the trial for each group | |
| Baseline data | 25 | A table showing baseline demographic and clinical characteristics for each group | 24 |
| Numbers analysed, outcomes and estimation | 26 | For each primary and secondary outcome, by group:<br>● the number of participants included in the analysis<br>● the number of participants with available data at the outcome time point<br>● result for each group, and the estimated effect size and its precision (such as 95% confidence interval)<br>● for binary outcomes, presentation of both absolute and relative effect size | |
| Harms | 27 | All harms or unintended events in each group | |
| Ancillary analyses | 28 | Any other analyses performed, including subgroup and sensitivity analyses, distinguishing pre-specified from post hoc | 17 |
| **Discussion** | | | |
| Interpretation | 29 | Interpretation consistent with results, balancing benefits and harms, and considering other relevant evidence | 8 - 11 |
| Limitations | 30 | Trial limitations, addressing sources of potential bias, imprecision, generalisability, and, if relevant, multiplicity of analyses | 13 |

Citation: Hopewell S, Chan AW, Collins GS, Hróbjartsson A, Moher D, Schulz KF, et al. CONSORT 2025 Statement: updated guideline for reporting randomised trials. BMJ. 2025; 388:e081123.
https://dx.doi.org/10.1136/bmj-2024-081123
© 2025 Hopewell et al. This is an Open Access article distributed under the terms of the Creative Commons Attribution License (https://creativecommons.org/licenses/by/4.0/), which permits unrestricted use, distribution, and reproduction in any medium, provided the original work is properly cited.

*We strongly recommend reading this statement in conjunction with the CONSORT 2025 Explanation and Elaboration and/or the CONSORT 2025 Expanded Checklist for important clarifications on all the items. We also recommend reading relevant CONSORT extensions. See www.consort-spirit.org.
